# Adverse Effects of Immune-Checkpoint Inhibitors: A Comprehensive Imaging-Oriented Review

**DOI:** 10.3390/curroncol30050355

**Published:** 2023-05-03

**Authors:** Carlo Augusto Mallio, Caterina Bernetti, Laura Cea, Andrea Buoso, Massimo Stiffi, Daniele Vertulli, Federico Greco, Bruno Beomonte Zobel

**Affiliations:** 1 Department of Medicine and Surgery, Fondazione Policlinico Universitario Campus Bio-Medico, 00128 Rome, Italy; c.bernetti@unicampus.it (C.B.); laura.cea@unicampus.it (L.C.); andrea.buoso@unicampus.it (A.B.); massimo.stiffi@unicampus.it (M.S.); daniele.vertulli@unicampus.it (D.V.); b.zobel@policlinicocampus.it (B.B.Z.); 2 Department of Medicine and Surgery, Research Unit of Radiology, Università Campus Bio-Medico di Roma, 00128 Roma, Italy; 3 Unità Operativa Complessa Diagnostica per Immagini Territoriale Aziendale, Cittadella della Salute Azienda Sanitaria Locale di Lecce, Piazza Filippo Bottazzi, 73100 Lecce, Italy; federicogreco@outlook.com

**Keywords:** adverse effects, imaging, cancer, immune-checkpoint inhibitors, immunotherapy

## Abstract

Immune-checkpoint inhibitors (ICIs) are immunomodulatory monoclonal antibodies, which increase antitumor immunity of the host and facilitate T-cell-mediated actions against tumors. These medications have been used in recent years as a weapon against advanced stage malignancies, such as melanoma, renal cell carcinoma, lymphoma, small or non-small cell lung cancer, and colorectal cancer. Unfortunately, they are not free from possible adverse effects (immune-related adverse events—irAEs) that mainly affect skin, gastrointestinal, hepatic, and endocrine systems. Early diagnosis of irAEs is essential to correctly and rapidly manage patients, with ICIs suspension and therapies administration. Deep knowledge of the imaging and clinical patterns of irAEs is the key to promptly rule out other diagnoses. Here, we performed a review of the radiological signs and differential diagnosis, based on the organ involved. The aim of this review is to provide guidance to recognize the most significant radiological findings of the main irAEs, based on incidence, severity, and the role of imaging.

## 1. Introduction

In recent years, the advancement of knowledge of the immune system’s molecular mechanisms against cancer has allowed the clinical development of new immunological therapies for the treatment of tumors, including checkpoint inhibitor therapy (ICIs). ICIs are antibodies that increase the antitumor immunity of the host, blocking inhibitors of T cell activation and function, such as T receptors, defined immuno-checkpoints, and facilitating T-cell-mediated actions against tumors. Inhibitors of checkpoints hold the function of restoring the normal action of T lymphocytes and, therefore, their ability to kill cancer cells, blocking the signal pathway of immunosuppressants. They work not only by stimulating T cells but also by switching on other innate and adaptive arm cells. 

The main targets include Cytotoxic T cells 4 (CTLA-4), receptor 1 of programmed cell death (PD-1), and its ligand (PD-L1) known as Ipilimumab, Tremelimumab, Pembrolizumab, Nivolumab, Atezolizumab, Avelumab, Durvalumab, or Cemiplimab (Table 1). The CTLA-4 and PD-1 receptors are negative regulators of the immune function of T cells; the inhibition of these targets consequently determines the increased activation of the immune system. These drugs are used in several advanced-stage malignancies, such as melanoma, renal cell or urothelial carcinoma, lymphoma, small or non-small cell lung cancer, and colorectal cancer, often improving the patients’ survival that, nevertheless, depends also on multiple variables such as the tumor histotype, the stage of disease, and clinical status of the patient [1,2,3]. At the same time, they can induce autoimmune toxicities with their mechanism of action, and it is important for the clinician to recognize their side effects, but also for the radiologist because sometimes imaging findings may precede clinical manifestation. 

Immune-related adverse events (irAEs) occur mainly in the skin, gastrointestinal, hepatic, and endocrine systems, including itching, rash, nausea, diarrhea, and thyroiditis (Table 2). The latter events are more frequently associated with treatment with anti-PD-1 antibodies (Pembrolizumab and Nivolumab), while colitis and hypophysitis with anti-CTLA-4 antibodies (Ipilimumab). The onset is typically between 3 and 6 months from initial administration; however, irAEs can occur at any time, even after cessation of treatment (“delayed effect”) [4].

A grading system called CTCAE v5.0 (Common Terminology Criteria for Adverse Events) has been created to classify irAEs into five grades based on symptoms, signs, or test results [5]. Grade 1 is generally asymptomatic or associated with mild symptoms without the need for intervention. Grade 2 usually requires minimal or non-invasive intervention. Whereas grade 3 is a severe medical condition in which hospitalization is indicated. Finally, grade 4 requires urgent intervention to avoid fatal consequences, and grade 5 corresponds to death determined by adverse effect to irAEs. The management of these adverse reactions can lead to a delay in the path of the patient’s oncological care. For instance, in some cases it is required to stop treatments, start steroid administration, or even hospitalize the patient. 

The incidence and severity or irAEs also depend on the agent; in fact, anti-CTLA4 usually has a greater percentage of adverse effects, even up to 80%, compared to anti-PD1 (27%) and anti-PDL1 (17%) [6].

In a review and meta-analysis, Wang et al. reported an incidence of adverse effects after PD-1 and PD-L1 inhibitors of 66%, with a grade 3 or higher in the CTCAE v5.0 of 14%. The reported incidence of serious and dangerous events is about 6% [7].

irAEs could be completely asymptomatic or paucisymptomatic. Tirumani et al., in a study regarding Ipilimumab therapy, reported an earlier onset of irAE radiological findings compared to their clinical manifestations, in more than 33% patients. Imaging techniques, such as Computed Tomography (CT), Magnetic Resonance Imaging (MRI), Ultrasound (US), or Fluorodeoxyglucose-Positron Emission Tomography (FDG-PET), usually carried out for oncological follow-up, can also recognize immune-related adverse event radiological findings, before the onset of clinical manifestations, allowing the classification of these conditions [8].

Hence, radiologists should be ready to identify the main imaging findings of irAEs, in order to discuss in a multidisciplinary team the more correct therapeutic management, commonly performed with ICIs suspension and corticosteroid administration. 

This is a comprehensive review of the main imaging features of irAEs, involving the colon, liver, pancreas, lung, endocrine system, kidney, heart, and nervous system, that could be a useful diagnostic tool for radiologists, but also for clinicians.

**Table 1:** Mechanism of action of the most-used ICIs and their more common indications [9,10]. Furthermore, there are other potential targets and mechanisms of action, some still under clinical trials, such as antibody anti-BTLA, VISTA, TIM-3, and CD47, agonist of costimulatory receptors (CD137, CD134, GITR, ICOS, CD40, and CD28). Other possible mechanisms of action include Chimeric Antigen Receptor T cell, adoptive cell transfer (ACT) of ex vivo expanded tumor-infiltrating lymphocytes (TILs), or ImmTAC (Immune Mobilizing Monoclonal TCRs Against Cancer). Moreover, oncolytic viruses (OVs) are another promising class of immunotherapies for treating cancer [11,12].

ICIs	Mechanism of Action	Indication
Ipilimumab [13]	Anti-CTLA-4 antibody	Melanoma Lung Ovaries Prostate Kidney Colorectal cancer
Tremelimumab [14]	Human IgG2 monoclonal antibody that blocks CTLA-4	HCC NSCLC
Pembrolizumab [15]	Anti-PD-1 antibody	Melanoma NSCLC and SCLS SCCHN Gastric and esophageal cancer Cervical cancer HCC Merkel cell carcinoma RCC Endometrial carcinoma Classical Hodgkin lymphoma Advanced Merkel-cell carcinoma Primary mediastinal large B-cell lymphoma Urothelial carcinoma
Nivolumab [13]	Anti-PD-1 antibody	Melanoma NSCLC and SCLC SCCHN RCC Urothelial cell carcinoma Classical Hodgkin lymphoma HCC CRC
Atezolizumab [7]	Anti-PD-L1 antibody	Urothelial cell carcinoma NSCLC and SCLC Breast cancer
Avelumab [16]	Anti-PD-L1 antibody	Merkel cell carcinoma Urothelial carcinoma RCC
Durvalumab [4]	Anti-PD-L1 antibody	Urothelial carcinoma NSCLC
Relatlimab [17]	Anti-LAG3 antibody	Melanoma

NSCLC: Non-Small-Cell Lung Cancer, SCLS: Small-Cell Lung Cancer, SCCHN: Squamous Cell Carcinoma of Head and Neck, HCC: Hepatocellular Carcinoma, RCC: Renal Cell Carcinoma, CRC: Colo-rectal Cancer, CTLA-4: Cytotoxic T cells 4, PD-L1: receptor 1 of programmed cell death (PD-1) and its ligand (PD L1).

**Table 2:** Main adverse effect organized according to the organ or system involved [18].

Organs	Symptoms
Systemic Adverse Effects	Fatigue Fever Anaphylaxis
Skin [19,20]	Pruritus Morbilliform or acneiform eruption Lichenoid reactions Eczema Vitiligo Psoriasis Bullous dermatoses Stevens–Johnson syndrome/toxic epidermal necrolysis (SJS/TEN) Drug reaction with eosinophilia and systemic symptoms (DRESS)
Intestinal [21,22]	Colitis Gastritis
Hepatic [23]	Hepatitis
Pulmonary [24]	Pneumonitis Pulmonary sarcoidosis Sarcoid-like granulomatous reactions
Endocrine system [25]	Hyper/hypo-thyroidism or thyroiditis Hypophysitis Diabetes Adrenal insufficiency
Cardiac [26]	Myocarditis Pericarditis Cardiomyopathy Cardiac fibrosis Arrhythmias
Renal [27]	Acute kidney injury (AKI) Immune-related renal disease
Rheumatologic [18]	Arthralgia Syndromes resembling rheumatoid arthritis and seronegative spondyloarthropathies Sicca syndrome Polymyalgia rheumatica Inflammatory myopathies Temporal arteritis and other vasculitis
Nervous system [28]	Neuropathy Myelopathy Guillain–Barré syndrome Myasthenia gravis Encephalitis/meningitis Posterior reversible encephalopathy syndrome
Ocular [18]	Uveitis, Peripheral ulcerative keratitis Vogt–Koyanagi–Harada syndrome Choroidal neovascularization Melanoma-associated retinopathy Idiopathic orbital inflammation Episcleritis, blepharitis Optic nerve swelling
Hematologic [18]	Aplastic anemia Autoimmune hemolytic anemia Immune thrombocytopenic purpura Neutropenia Acquired hemophilia A Cryoglobulinemia

## 2. Gastrointestinal

### 2.1. Incidence

After dermatitis, GI tract toxicity is the most frequent irAEs in patients treated with ICIs, with a higher incidence and worst severity in cases of treatment with anti–CTLA-4 antibodies (30–40%) or a combination of different ICIs (10%) [21,29].

ICI-related inflammation can involve any portion of the alimentary canal, from oral mucosa to rectum; hence, inflammation of the upper GI tract can also happen with esophagitis, gastritis and, duodenitis [22,30,31].

Upper GI adverse events, more commonly related to PD-1 inhibitors, are usually less frequent and poorly described in the literature. Conversely, lower GI adverse events are reported in almost 1/3 of patients treated with CTLA-4 inhibitors. 

### 2.2. Sign and Symptoms

This kind of toxicity more commonly involves small and large bowels, presenting usually with enterocolitis, mainly because of CTLA4 therapy (Ipilimumab). Ileitis without colitis is, instead, an uncommon event. 

Colitis may present with a wide spectrum of manifestations; it could also be asymptomatic. However, the most frequent symptoms are abdominal pain and diarrhea, that reach a reported incidence of almost 45% in cases of combined ICI therapy [13,32].

Fever, hematochezia, or mucus in stool and endoscopic evidence of colon inflammation could be present [13]. Upper GI toxicity can be an isolated event, but it frequently coexists with lower GI toxicity, usually presenting with decreased appetite, abdominal pain, and nausea and/or vomit, which are non-specific symptoms often encountered in cancer patients [33]. 

Severe cases that implicate life-threatening complications, such as bowel perforation, sepsis, bleedings, and dehydration, are rare. 

The symptoms usually occur after the second or third dose of ICI, within 6–8 weeks from the beginning of treatment; nevertheless, they could manifest with various onset timings even several months after the end of therapy (“delayed toxicity”) [32].

### 2.3. Diagnosis and Imaging

Diagnosis of upper or lower GI involvement is achieved with endoscopy, which can demonstrate inflammation, erythema, ulceration, and mucosal friability. Sometimes a histological sampling is performed, showing neutrophilic, lymphocytic, or eosinophilic intra-epithelial infiltrates and crypt invasion. 

Nevertheless, CT could offer a less invasive, though less accurate, diagnostic alternative; moreover, in some cases, imaging features could precede the onset of symptoms [34].

In two studies, Garcia-Neur et al. and Tirumani et al. have reported a good correlation between CT findings and evidence of ICIs-related colitis. In a study aimed at recognizing colitis, involving 53 patients, Wang et al. reported a sensitivity of CT of 53% and a specificity of 78% [35].

Furthermore, CT should always be considered if severe complications are suspected, for instance toxic megacolon, bleedings, or intestinal perforation [34]. 

**Figure 1:**
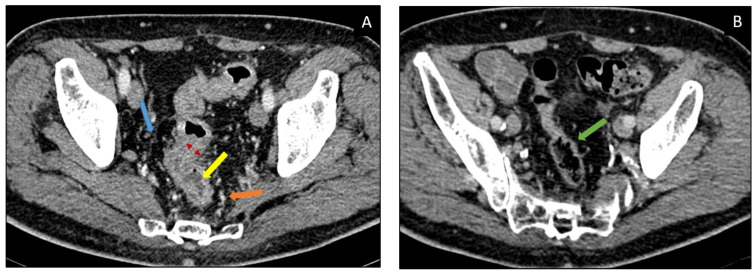
73-year-old patient with lung squamous cell carcinoma under Pembrolizumab. (**A**) Axial post-contrast CT image demonstrating colitis after 8 weeks of ICI administration, with diffuse bowel wall thickening (red arrowheads) and mucosal hyperemia (yellow arrow), at the level of sigma and rectus; also associated with peri-visceral fat stranding (blue arrow) and local vascular engorgement (orange arrow). (**B**) Axial post-contrast CT image showing colitis resolution (green arrow) after Pembrolizumab discontinuation and corticosteroid administration.

There are two main CT patterns of immune-related colitis described in the literature, diffuse colitis or segmental colitis associated with diverticulosis (SCAD) which is usually confined to the sigmoid (Figure 1). A less frequent pattern has been described by Barina et al. which corresponds to isolated recto-sigmoid colitis without diverticulosis (Table 3) [36].

### 2.4. Differential Diagnosis

Possible differential diagnoses are Crohn’s disease and ulcerative colitis, infectious colitis, and pseudomembranous colitis. 

### 2.5. Management

GI adverse effects management is performed by discontinuing ICI administration and starting methylprednisolone; Infliximab or Vedolizumab may be considered as a second-line therapy. In grade 4, the permanent withdrawal of all ICIS is strongly recommended. 

## 3. Liver

### 3.1. Incidence

A common irAEs is represented by immuno-related hepatotoxicity (IRH), which occurs in almost 30% of patients. Incidence is usually higher in CTLA-4 antibody therapy (15%), and even higher in cases of combined therapy with PD-1/PD-L1-blocking antibodies (30%) [39].

### 3.2. Sign and Symptoms

Generally, patients are asymptomatic. Alterations in laboratory values may be observed, for instance mild elevation of ALT and AST or bilirubin. The most common symptoms are fever, abdominal pain, weakness, nausea, vomiting, and jaundice [23,39]. Fulminant hepatitis is an extremely rare event, happening in less than 1% of cases (<0.2%) [39,40]. It may present as a hepatitis pattern with hepatocellular injuries or a cholangitic pattern with predominant bile duct involvement, which is less common. The mean time of onset is usually between 3 and 14 weeks after the beginning of ICI therapy [39].

### 3.3. Diagnosis and Imaging [16,41,42,43,44]

IRH more commonly present with peri-portal edema, accompanied by lymphadenopathies and ascites. In the following table (Table 4) we reported the main findings in different diagnostic modalities.

### 3.4. Differential Diagnosis

Differential diagnosis includes idiopathic autoimmune hepatitis, viral hepatitis, and alcoholic liver disease [45]. 

### 3.5. Management 

Treatment consists of the suspension of ICI and administration of corticosteroids. Mycophenolate Mofetil is an alternative for non-responsive patients. 

## 4. Pancreas

### 4.1. Incidence

ICI-induced pancreatic injury (ICIPI) is a less common condition, which can involve both the endocrine and exocrine pancreatic function [46,47]. 

George et al. reported, in a large meta-analysis, a lower incidence of lipase elevation in patients treated with monotherapy with PD-1 inhibitors or CTLA-4 inhibitors (<5%) and a higher incidence in cases of combined therapy (14%) [21]. Lipase elevation usually rises between 9 to 20 weeks, according to Abu-Sbeih et al. [48]. 

ICI-associated acute pancreatitis is rare, with an incidence of less than 1%. Chronic pancreatic dysfunction may occur. No deaths due to ICI-associated pancreatitis have been reported to date [48].

### 4.2. Sign and Symptoms

ICIPI initially could present only with a transient asymptomatic elevation in serum lipase or amylase, which, however, are not routinely monitored [42].

Clinically, ICIPI may manifest with a wide spectrum of symptoms. The most common manifestation corresponds to irregular stools with diarrhea, weight loss, and a transient asymptomatic serum amylase and lipase increase [22]. It can present as endocrine or exocrine pancreatic insufficiency, acute and chronic pancreatitis, but also an autoimmune pancreatitis pattern has been reported. Acute pancreatitis is rare and usually presents after an average of 4 months from the initiation of therapy [46,48].

Both these entities, pancreatitis and the isolated increase of pancreatic enzymes, are stratified according to severity, using the latest version of CTCAE. 

### 4.3. Diagnosis and Imaging

**Figure 2:**
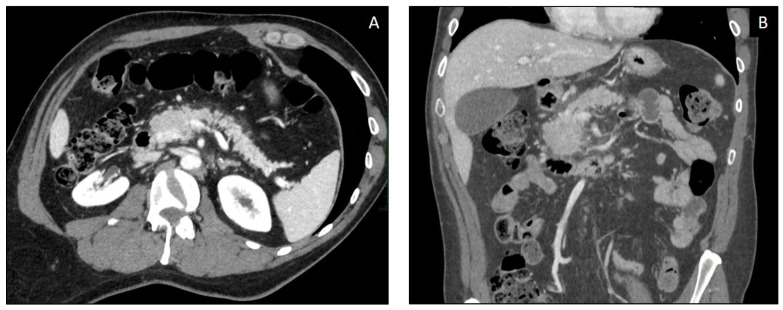
50-year-old patient with melanoma treated with Ipilimumab. (**A**,**B**) Axial and coronal post-contrast CT images, demonstrating acute pancreatitis after 10 weeks of ICI administration, with parenchymal enlargement and inhomogeneous enhancement with diffuse hypodensity at the level of the head and uncinate process, associated with adjacent fat stranding.

Even though imaging characteristics of pancreatic involvement during ICI therapy are still not well described in the literature, it may manifest as traditional acute pancreatitis [46,49]. Diagnosis of this condition requires two of the following three criteria: epigastric pain, elevated serum lipase (>3 times the upper normal limit), and specific imaging findings [43]. It can present either as necrotizing pancreatitis (NP) or as interstitial edematous pancreatitis (IEP), the latter being the most frequent imaging finding of ICIPI (Figure 2) [50].

There are two reported recognizably different radiological patterns of pancreatic inflammation: acute interstitial pancreatitis with focal or diffuse pancreatic enlargement with heterogenous enhancement, segmental hypoenhancement, and peripancreatic fat-stranding (80%); or autoimmune pancreatitis pattern, which usually presents with a sub-acute onset, as a mass-like enlargement (“sausage shaped pancreas”) without peri-pancreatic edema and fat-stranding (16%) (Table 5) [46,50]. 

ICIPI may also manifest as chronic pancreatitis which can evolve in parenchymal atrophy, often leading to long-term complications such as diabetes mellitus [51].

**Table 5:** Imaging findings of ICI-related pancreatitis [46,50,52].

Imaging Modality	ICIPI Imaging Findings (IEP > NP)
US	Limited role (hindered by patient’s habitus and bowel gas) - Initial diagnostic workup: to exclude biliary stones or biliary tree dilatation - Follow-up: re-evaluation of complications Edematous pancreatic regions (focal or diffuse): Pancreatic enlargement Reduced echogenicity
CT	IEP Focal, diffuse, or mass-like pancreatic enlargement, consequent to inflammation and edema, and either focal, diffuse, or heterogeneous decrease in the parenchymal enhancement. Peri-pancreatic fat stranding NP Necrotic areas involving pancreatic gland and/or peripancreatic spaces: well-defined, hypodense, inhomogeneous, non-enhancing area Early complications (<4 weeks): Acute peri-pancreatic fluid collections (APFCs): in the peri-pancreatic space Hypodense homogeneous, due to the presence of fluid
MRI	IEP With edematous pancreatic regions: T1w pre-contrast: hypointense T2w: slightly hyperintense DWI: restricted diffusion NP with necrotic areas involving pancreatic gland and/or peripancreatic spaces: T1w pre-contrast: hypointense T2w: hyperintense Early complications (<4 weeks) Acute peripancreatic fluid collections (APFCs): in the peripancreatic space T2w: hyperintense T1w pre-contrast: hypointense T1w post-contrast: no enhancement Acute necrotic collections (ANCs): In pancreas or in peripancreatic space Heterogeneous content due to the presence of debris in addition to fluid Delayed complications (>4 weeks) Both APFCs and ANCs become encapsulated, with a uniform enhancing capsule containing only fluid (pseudocyst) or fluid, debris, and loculations (walled-off necrosis). Necrotic areas may appear more inhomogeneous because of superinfection.
PET-CT (18F-FDG)	Focal * or diffuse radiotracer uptake

* Can mimic malignant lesions. ICIPI: ICI-induced pancreatic injury, US: Ultrasound, CT: Computed Tomography, MRI: Magnetic Resonance, PET: Positron Emission Tomography, FDG: fluorodeoxyglucose, NP: necrotizing pancreatitis, IEP: Interstitial Edematous Pancreatitis, T1w: T1 weighted images, T2w: T2 weighted images, DWI: Diffusion Weighted Imaging, APFCs: Acute peri-pancreatic fluid collections, ACNs: Acute Necrotic Collections.

### 4.4. Differential Diagnosis

Differential diagnosis is challenging, and it is usually between ICI-related pancreatitis and immunoglobulin G4-related autoimmune pancreatitis. The onset of the latter is more commonly acute, with upper abdominal pain and obstructive jaundice. Imaging of immunoglobulin G4-related autoimmune pancreatitis shows a typical appearance of “sausage pancreas”, with loss of normal fatty lobulations. Simultaneous findings in other organs, for instance biliary, salivary, aortic, and retroperitoneal involvement, can be present [46,50].

### 4.5. Management

Prednisone and Methylprednisolone treatment is usually indicated in grades 3 and 4.

## 5. Lung

### 5.1. Incidence

Pneumonitis is one of the most frequent irAEs. The incidence of ICI-therapy-related pneumonitis is estimated to be between 3% and 6% [53]. It occurs mostly in patients receiving combined ICIs treatment (10%), in approximately 1–5% of patients treated with anti-PD-1/PD-L1 agents, and in less than 1% of patients treated with anti–CTLA-4 antibody [54].

Naidoo et al. evaluating the incidence of pulmonary irAEs in different lung tumors treated with anti-PD-1/PD-L1, as monotherapy or in combination with anti–CTLA-4, reported a greater likelihood of developing these conditions in cases of hematologic malignancies (4/35—11%), breast carcinoma (1/14—7%), and head and neck squamous carcinoma (1/10—10%) [24,55].

### 5.2. Sign and Symptoms

The mean time of onset of ICI-related pneumonitis is about 2 to 3 months after beginning the therapy, or even earlier in cases of combined therapy or with non-small cell lung cancer (NSCLC).

Sometimes patients may be either asymptomatic, and diagnosis is made only based on laboratory results or CT, or they may present with mild to severe symptoms and signs such as dry cough, fever, chest pain, shortness of breath, and fine inspiratory crackles. 

### 5.3. Diagnosis and Imaging and Differential Diagnosis

**Figure 3:**
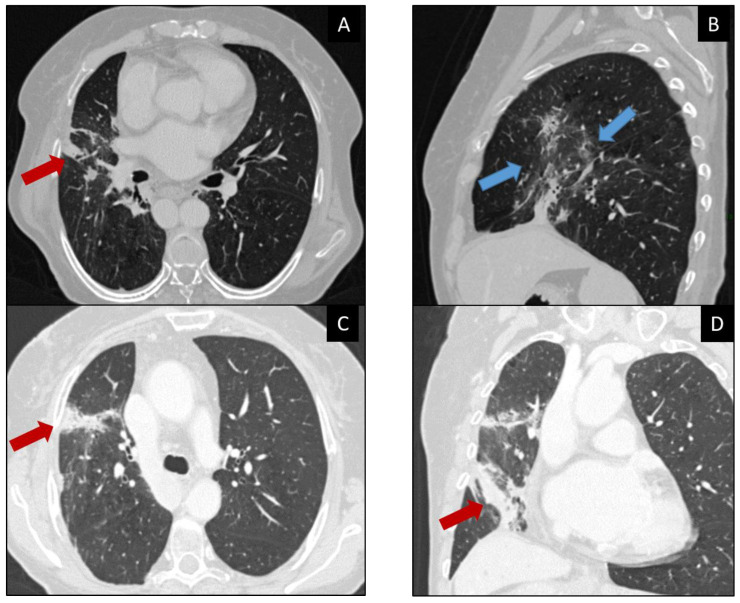
65-year-old patient with history of lung adenocarcinoma (not shown in the images) under Nivolumab. Axial, sagittal, and coronal CT images of the chest (**A**–**D**) showing mixed abnormalities of the right lung with consolidations (red arrows) and diffuse ground-glass opacities (blue arrows). ICI-related pneumonitis was confirmed by clinical and laboratory data. Corticosteroids were administered and Nivolumab therapy was suspended with improvement of the clinical picture.

In clinical and anamnestic suspect of immune-related pneumonitis, a chest CT scan is necessary. CT can generally show ground-glass pattern and/or disseminated nodular infiltrates, most often in the lower lobes (Figure 3) [56].

Sometimes it might be useful to also conduct a spirometry and a bronchoscopy with bronchoalveolar lavage to rule out infectious agents which in most cases are Pneumocystis Jirovecii and respiratory viruses such as influenza, metapneumovirus, or the syncytial virus B [56]. 

Four CT patterns of pneumonitis have been described by Nishino et al. identified in patients treated with PD-1 inhibitors: cryptogenic organizing pneumonia (COP), which is the most common; non-specific interstitial pneumonia (NSIP); hypersensitivity pneumonitis (HP); and acute interstitial pneumonia/acute respiratory distress syndrome (AIP/ARDS) (Table 6) [57].

In 2021, a case report written by Kucukarda et al. showed two cases of secondary immune-related pneumothorax based on immune pneumonitis, respectively, in a 25-year-old woman treated with Atezolizumab, with spontaneous resorption of the pneumothorax, and a 36-year old female patient treated with Nivolumab in which therapy was discontinued permanently [58].

Sometimes, such as in non-small cell lung cancer (NSCLC), the evaluation of CIP (checkpoint inhibitor pneumonitis) may be more complicated due to overlap with radiological finding of the primary tumor [59].

The bronchiolitis pattern is rare and the presentation is often non-specific. 

It is worth mentioning two other lung manifestations associated with ICIs: sarcoid-like reaction and radiation recall pneumonitis. 

Sarcoid-like reaction occurs generally in patients affected by melanoma treated with Ipilimumab (anti-CTLA-4) in a percentage of 5–7% [54,60].

It is characterized by a focal area of consolidation in the upper lobes of lungs, associated with symmetric hilar and mediastinal lymphadenopathy [60]. Histologic examination can be useful to differentiate metastatic lesions.

Radiation recall pneumonitis is a rare condition occurring in previously irradiated areas of pulmonary tissue after the application of triggering agents (e.g., immunomodulators). Findings include consolidative or ground glass alterations.

### 5.4. Management

In moderate form, systemic steroid administration is the first therapeutic step. In severe cases Infliximab and/or admission to the intensive care unit can be necessary [59].

## 6. Endocrine System

Immune-related endocrine adverse events are among the most common toxicities in patients treated with ICIs, happening in almost 40% of cases [61,62]. 

In a meta-analysis by Abdel-Rahman et al., patients treated with these therapies reported a greater risk of hypophysitis, hypo- and hyperthyroidism, and adrenal insufficiency, but without a specific association with incidence, severity, and distinct tumors or drugs (CTLA-4 vs. PD-1) [63]. 

Other studies demonstrated a greater prevalence of thyroid disorders in the case of anti-PD-1 treatments and a higher rate of pituitary disorders in patients treated with anti CTLA-4 [63]. 

In descending order, the most common organs involved are thyroid, pituitary, adrenal, and beta cells of pancreatic islets. 

Even though symptoms usually present within 6 months of ICI initiation, the onset can be variable and unpredictable [64]. 

Severity is widely variable. Typically, these endocrinopathies are manageable with prompt recognition and treatment; however, they are rarely fatal, but rather these conditions can alter the patients’ quality of life. In fact, most other systems are affected by transient inflammation that resolves with the restoration of normal organ function, whereas ICI-related endocrinopathies can result in permanent, irreversible endocrine dysfunction [65]. Some cases of diabetes mellitus (DM) have proven fatal when presenting in diabetic ketoacidosis.

Manifestations are usually subtle and non-specific, often overlapping with cancer-related or other therapy-related complications (e.g., fatigue, nausea); hence, hormone level monitoring is critical.

## 7. Thyroid

### 7.1. Incidence

Thyroid toxicity is more frequent in women, mostly in patients treated with combined immunotherapy PD-1/CTLA-4 blockade (20%) and with anti-PD1/antiPD-L1 monotherapy (up to 10%); it is less common in anti-CTLA4 treatment (5%) [66]. 

Hypothyroidism, determined by destructive thyroiditis, is the most frequent manifestation, happening in 30–40% of anti-PD-1/PD-L1 induced cases [67].

### 7.2. Sign and Symptoms

Thyroid toxicity could present with hypothyroidism or thyrotoxicosis and it is usually asymptomatic. Symptoms of hypothyroidism are usually general, such as fatigue, weight gain, constipation, depression, and altered cognition, whereas, in cases of thyrotoxicosis, they are mainly agitation and palpitations. 

Thyroid storms are very rare. ICI-related Graves’ disease has been rarely reported. 

The median time to onset is 6 weeks after ICI initiation, although thyroid disfunctions may occur any time-point during therapy.

### 7.3. Diagnosis and Imaging

Thyroid function test alteration may be present, along with peroxidase and anti-thyroglobulin anti-thyroid antibodies, and should be monitored during ICI therapy to guarantee the appropriate diagnosis and treatment of thyroid toxicity [62]. 

In cases of clinical or laboratory suspect of thyroid dysfunction, US is the first imaging modality to adopt (Table 7) [68].

After symptoms have resolved the gland could appear hypotrophic, hypoenhancing, and non FDG-avid. 

### 7.4. Differential Diagnosis

Differential diagnosis may include thyroid metastasis, or, in cases of hypothyroidism, it could be a consequence of ICI-related hypophysitis. 

### 7.5. Management 

In the context of thyroid disfunction, the suspension of ICIs is not mandatory, and steroids may be proposed. Levothyroxine is the treatment of choice for hypothyroidism, whereas anti-thyroid drugs and beta-blockers should be considered for the management of hyperthyroidism [68].

## 8. Pituitary Gland

### 8.1. Incidence

ICI-related hypophysitis is a relatively common irAE, happening in almost 5% of patients treated with ICIs. Incidence is generally higher in cases of anti-CTLA4 treatments (13%), whereas it is a rare condition in cases of anti-PD1 and anti-PDL1 ICI treatments (1%) [69]. 

### 8.2. Sign and Symptoms

**Figure 4:**
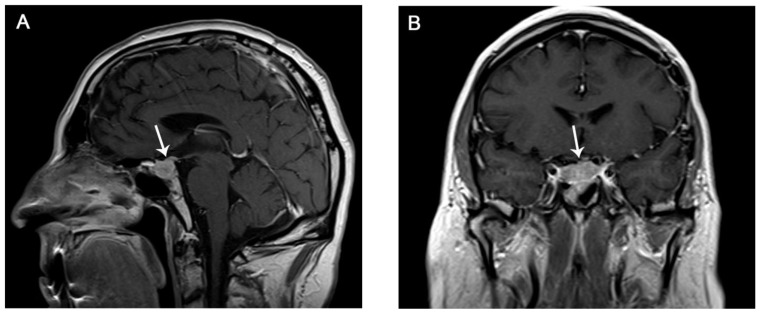
71-year-old male with melanoma, who, after several doses of Ipilimumab, developed fatigue, hallucinations, and laboratory findings of panhypopituitarism. Post-contrast T1-weighted MR sagittal (**A**) and coronal (**B**) images revealed enlargement of the pituitary gland and infundibulum with heterogeneous contrast enhancement (arrows in (**A**,**B**)). Ipilimumab was discontinued and steroid therapy was started with improvement on the clinical picture. Follow-up MRI was not available.

The median time of onset is 9 weeks, and the most common symptoms are headache, fatigue, and hypopituitarism-related symptoms (hypothyroidism, hypogonadism, and hypocortisolism) [70]. Severe conditions, such as life-threatening acute panhypopituitarism, are rare (Figure 4) [25].

### 8.3. Diagnosis and Imaging

**Figure 5:**
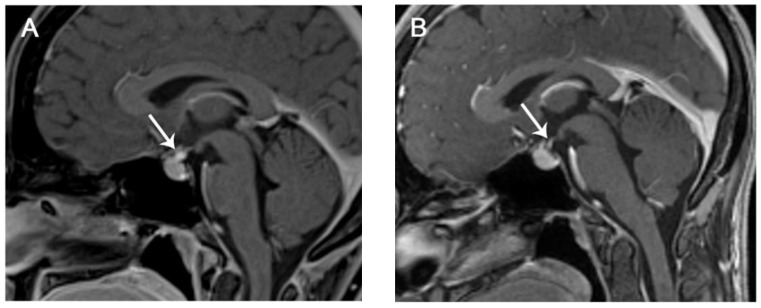
35-year-old with melanoma treated with Ipilimumab. After the third dose, she developed headache and nausea. Post-contrast T1-weighted MR sagittal images obtained at onset (**A**) and at the 6-month follow-up (**B**). Subtle thickening of the pituitary stalk is seen at the first MRI (arrow in (**A**)). Post-contrast T1-weighted MR image after discontinuation of Ipilimumab and application of steroid therapy showed normalization of the pituitary stalk (arrow in (**B**)), suggesting mild infundibular hypophysitis.

Hypophysitis can be asymptomatic, and, hence, could be incidentally detected with imaging before it becomes clinically evident (Figure 5) (Table 8). 

However, if suspected, hormones should be measured and the programmed dose of ICI should be withheld. Moreover, an MRI should always be performed to confirm diagnosis and exclude mimickers, such as metastasis and pituitary adenoma [71,72].

Clinical and laboratory monitoring is pivotal for the correct diagnosis because a normal hypophysis on imaging does not rule out hypophysitis [73].

### 8.4. Differential Diagnosis

Differential diagnosis includes other disorders such as pituitary adenoma, metastasis, and lymphocytic hypophysitis (Table 9) [71,73]. 

### 8.5. Management

Management of this condition is usually performed by suspending ICIs and starting hormone replacement therapy (HRT) associated in some cases with steroids [73].

## 9. Adrenal Gland

### 9.1. Incidence

Incidence of ICI-related adrenalitis is reported to be less than 5% in patients treated with Nivolumab or Pembrolizumab [62,72,74].

### 9.2. Sign and Symptoms

This condition usually presents with adrenalitis and the related hormone insufficiency; however, it may also be a consequence of hypopituitarism. 

Symptom’s severity is variable, ranging from aspecific clinical manifestations, such as fatigue, to adrenal crisis, which requires prompt intervention. 

### 9.3. Diagnosis and Imaging

If ICI-related adrenalitis is clinically suspected, ACTH, cortisol, electrolytes, aldosterone, and renin must be dosed and strictly monitored. Imaging could be non-specific, manifesting only bilateral enlargement and mild uptake in 18F-FDG PET-CT [75].

### 9.4. Differential Diagnosis

Adrenal enlargement due to ICIs administration could be mistaken for metastasis, in particular in non-small cell lung cancers or melanoma which are common in malignancies treated with ICIs [76]. 

### 9.5. Management

In cases of adrenal insufficiency, prompt hospitalization and endocrinologic consultation may be necessary to allow volume replacement and the immediate intravenous administration of glucocorticoids [62,74,77].

## 10. Endocrine Pancreas

### 10.1. Incidence

Pancreatic endocrine toxicity has been reported in less than 1% of patients, mostly related with PD-1/PD-L1 antibodies and in 1.5% of patients treated with combination CTLA-4/PD-1 therapy [78].

ICI-related diabetes’ underlying mechanism is believed to be autoimmune, since GAD65 antibodies and the reduced expression of PD-1 on T cells have been observed, determining an autoimmune response against pancreatic B cells [51,79].

Hyperglycemia may also occur after corticosteroids administration, commonly used to treat some irAEs, such as pneumonitis or enterocolitis [72]. 

### 10.2. Sign and Symptoms

It may manifest itself with various degrees of hyperglycemia, diabetes mellitus; rarely may it begin with ketoacidosis. The onset is variable, usually ranging from 1 month to 1 year [63,79].

### 10.3. Diagnosis and Imaging 

Pancreatic endocrine toxicity usually manifest with a “sausage-like” pancreatic enlarment with loss of pancreatic lobulations. In Table 10 we reported the main radiological findings reported in different diagnostic modalities.

### 10.4. Differential Diagnosis

Pancreatic malignancies and inflammatory conditions non-related to therapy are the main differential diagnosis [50,79]. 

### 10.5. Management

Insulin administration is the therapy of choice in cases of hyperglycemia and suspected ketoacidosis, along with ICI suspension. Blood tests, more specifically periodic fasting plasma glucose and hemoglobin A1c, should be routinely performed and monitored in cases of PD-1/PD-L1 inhibitors therapy [65,66].

## 11. Renal Toxicity

### 11.1. Incidence

Another less commonly encountered irAE corresponds to nephrotoxicity, which has been described to be 5% in cases of combined treatments, and as a rare event in monotherapy (<1%). The median time of developing typically is within 3–6 months after the first dose of ICIs [27,80].

### 11.2. Sign and Symptoms

ICIs-induced kidney damage is mostly as acute kidney injury (AKI). Other types of renal injury were only reported in case reports, such as nephrotic syndrome without AKI or renal tubular acidosis [27].

### 11.3. Diagnosis and Imaging

Sometimes the only sign of kidney injury is the asymptomatic increase in serum creatine, which is also an indicator of the degree of severity (Table 11). 

### 11.4. Differential Diagnosis

AKI, also known as acute renal failure (ARF), may be associated with multiple causes that are generally divided into prerenal (diminished renal blood flow), direct damage to the kidneys, and urinary tract obstruction. 

### 11.5. Management

In cases of values of creatinine greater than 3times the baseline value or >4.0 mg/dL, hospitalization is indicated with definitive withdrawal of ICIs combined with the administration of corticosteroids. In severe cases, dialysis could be started. 

## 12. Cardiac Toxicity

### 12.1. Incidence

Cardiotoxicity represents an uncommon irAE (incidence < 1%). Nevertheless, the mortality rate is high (50%); therefore, clinicians and radiologists need to be aware of these complications and to recognize the main signs, symptoms, and imaging findings, to promptly achieve the diagnosis and appropriately guide patient management. 

Cardiovascular irAEs are more common in patients treated with ICI in combinations than in those undergoing monotherapy. Moreover, they are more likely in cases of co-administration of another cardiotoxic drug or in cases of previous cardiovascular disease or pre-existing risk factors. 

The most reported complications are myocarditis, with a higher incidence in the case of combined treatment with Nivolumab and Ipilimumab [81].

### 12.2. Sign and Symptoms

Clinical symptoms and signs of ICI-related myocarditis are heterogeneous; hence, diagnosis is often challenging. In addition, pathophysiology is still not completely understood. Studies suggest that a tumor-activated T cell population may cross-react with cardiac antigens exposed on cardiomyocytes. It can occur from 2 days to 15 months after the start of ICIs and is potentially fatal [26].

### 12.3. Diagnosis and Imaging

The gold standard for the diagnosis of myocarditis is the endomyocardial biopsy, which, however, as an invasive approach is accomplished in rare cases. On the other side, cardiac biomarkers and ECGs are non-specific although easy to use. Therefore, the diagnostic challenges have opened the door to different imaging techniques in assessing ICI-induced cardiotoxicity (Table 12).

### 12.4. Differential Diagnosis

Myocarditis has many different etiologies, such as infectious (viral, bacterial, fungal, or parasites) or non-infectious. Non-infectious etiologies include radiation, transplant rejection, giant cell myocarditis, autoimmune disease, and also drugs (for instance antibiotics, anticonvulsants, anti-inflammatories, or diuretics). 

### 12.5. Management

According to CTCAE v5.0 after grade 1, permanent withdrawal of ICIs is necessary. In some refractory cases, corticosteroids and Infliximab are recommended.

## 13. Neurological Toxicity

### 13.1. Incidence

The incidence rate of neurological irAEs (nAEs) in the literature accounts for about 1% to 10%; in particular, it is reported to be 4% for anti-CTLA, 6% for anti-PD-1, and 12% for combined immunotherapy [28,82]. Even though severe nAEs are rare (≤1%), they can lead to severe long-term neurological deficits and death; therefore, it is necessary a prompt diagnosis. 

### 13.2. Sign and Symptoms

NAEs can involve both the central and peripheral nervous system, the latter being more mild and more common [83]. 

Symptoms are often non-specific, such as headache, weakness, fatigue, vertigo, and dizziness, with a challenging diagnosis. However, this condition could also manifest as clinical syndromes such as myasthenia gravis, Guillain–Barré Syndrome, and transverse myelitis, and also with rare life-threatening conditions such as aseptic meningitis, encephalitis, and posterior reversible encephalopathy syndrome (PRES) [84].

Peripheral neuropathies, including focal and peripheral neuropathies, Guillain–Barré-like syndromes, and meningoradiculitis, are the most commonly described in the literature.

Onset is variable, from a few days to months, with a median time of 6 weeks [28].

### 13.3. Diagnosis and Imaging

Diagnosis of nAEs is usually challenging, due to the wide variety of clinical presentation, and should only be considered after exclusion of other routine causes such as infection, space occupying lesions, toxins, and metabolic and autoimmune disorders [82]. 

An adequate systemic assessment of the nervous system is the first step, followed by serological tests for infections, lumbar puncture, nerve conduction studies, and imaging [82,84].

If central nervous system toxicity is suspected CT and/or MRI should be performed together with diagnostic lumbar puncture, to rule out brain metastases, leptomeningeal disease, or inflammation. 

### 13.4. Differential Diagnosis

Differential diagnosis includes malignancies, infections, and metabolic alterations [82,84].

### 13.5. Management

Similar to other irAEs, CNS adverse events generally require the temporary or permanent discontinuation of ICIs and the administration of steroids. Nevertheless, some NAEs, for instance in ICI-related Myasthenia Gravis or Guillain–Barré syndrome, may need specific treatments, such as intravenous immunoglobulin or plasmapheresis [82]. 

## 14. Conclusions

ICIs have been and are used successfully to treat several advanced-stage malignancies. Despite being effective in treating cancer, they might trigger immune-related adverse events—irAEs, which can affect various organs with different degrees of severity, ranging from mild reactions to patient’s death. Adverse reactions to ICIs can be subtle and multifaceted; thus, a close collaboration between clinicians and radiologists is of paramount importance in dealing with this peculiar category of patients. Early diagnosis and rapid management are essential; therefore, a deep, transversal knowledge of co-therapeutic schemes, main signs, symptoms, and imaging patterns of irAEs is key to promptly solve diagnostic challenged posed by irAEs and eventually suspend ICIs and/or initiate the most appropriate therapy.

## Figures and Tables

**Table 3:** CT and PET-CT (18F-FDG) findings in GI irAEs [22,37,38].

CT Findings	Description
Bowel wall thickening Focal: extension < 5 cm Segmental: extension of 6–40 cm Diffuse: extension > 40 cm	Bowel wall 2 mm if lumen distended >5 mm if lumen collapsed
Mucosal hyperemia	Increased enhancement of the colonic mucosa compared to other GI tracts
Mesenteric vessel engorgement	Prominence or tortuosity of the vasa recta
Distended colon, fluid filled and air-fluid levels	Colon > 6 Cecum > 9 cm
Pericolic fat stranding	Abnormal increased attenuation in pericolic fat (ground-glass-like, reticular pattern, reticulonodular)
**PET-CT (18F-FDG)**	**Description**
FDG-uptake	Diffuse or segmental (possible false positive in patients in treatment with metformin; consider suspension 48 h before of the PET-CT)

CT: Computed Tomography, PET: Positron Emission Tomography-Computed Tomography, FDG: fluorodeoxyglucose.

**Table 4:** Imaging findings in Liver irAEs [22,36,39].

Imaging Modality	Findings
US, CT, MRI	Peri-portal edema - US: prominent echogenicity of portal vein walls or periportal spaces - CT: diffused or geographical areas of parenchymal hypoattenuation (may obscure or mimic liver metastases) - MR T2w: hyperintense signal of portal vein wall and of surrounding tissues Lymphadenopathy Peri-hepatic ascites Cholecystitis with wall thickening and edema (sometimes without imaging detectable gallstones) Hepatomegaly and steatosis
Cholangiopancreatography (or endoscopic US)	Extra-hepatic predominant cholangitis Extra-hepatic bile duct dilatation, usually without obstruction Intrahepatic predominant cholangitis Multifocal dilatation or narrowing of intrahepatic ducts
Biopsy with histologic analysis	Only method that allows differential diagnosis between hepatitis and other etiologies Can present with: Hepatitis pattern (hepatocellular injuries) Cholangitic pattern (predominant bile ducts injury, less common)

US: Ultrasound, CT: Computed Tomography, MRI: Magnetic Resonance.

**Table 6:** Imaging findings of ICI-related pneumonitis [46,50,52].

CT Patterns of Pneumonitis (in Order of Incidence)	CT Findings	Location	Differential Diagnosis
COP	Patchy GGOs Consolidation with bronchogram Nodule: small peribronchovascular or mass-like with spiculated margins (mimicking a malignancy) Occasionally accompanied by the reversed “halo sign” or atoll sign (round consolidative opacity surrounding a central ground-glass area)	Bilateral Lower lobes Sub-pleural or peri-bronchial	Clinical history and laboratory findings are essential Pneumonitis with different etiology: Drug-induced Radiation-related: Area of lung exposed at least to 30 gray Ground-glass opacities with possible increase in density over time Not limited by interlobar fissures or bronchovascular structures Infective (bacterial, viral—COVID-19, fungal—aspergillosis) Bacterial: consolidations with air-bronchogram and pleural effusion Cryptogenic
NSIP	GGOs Opacities Traction bronchiectasis or bronchiolectasis Minimal or absent honeycombing	Bilateral Lower lobes Sub-pleural sparing of the posterior lower lobes (DD from infections)	
HP	Centrilobular ground-glass nodules Mosaic attenuation due to air trapping	Upper lobe Centrilobular	
AIP/ARDSuncommon and severe	Geographic or diffuse GGOs Consolidation in the dependent lung Interlobular septal thickening (“Crazy paving”) Traction bronchiectasis Bronchiolitis: centrilobular nodules, with a tree-in-bud arrangement	Bilateral Dependent regions Ventro–dorsal and craniocaudal gradient	
Sarcoid-like reaction	Small nodules peribronchovascular (pulmonary granulomatosis) Symmetric hilar and mediastinal lymphadenopathy	Upper lobes	Histologic examination fundamental Metastatic lymphadenopathy and lung metastases

COP: Cryptogenic Organizing Pneumonia, NSIP: Non-Specific Interstitial Pneumonia, HP: Hypersensitivity Pneumonitis, AIP/ARDS: Acute Interstitial Pneumonia/Acute Respiratory Distress Syndrome (AIP/ARDS), DD: Differential Diagnosis, GGOs: Ground-Glass Opacities.

**Table 7:** Imaging findings of ICI-related thyroid toxicity [27].

Imaging Modality	Findings
US Similar to Hashimoto’s thyroiditis	Diffuse enlargement of the gland Heterogeneous and hypoechoic parenchyma, often with a nodular or pseudo nodular pattern Doppler: increased (normal or decreased) vascularity
TC	Diffuse enlargement of the gland Hypodensity C+: Hypoenhancement After switching from hyperthyroidism to hypothyroidism, the gland remains hypodense but decreases in size
PET-CT (18F-FDG)	Diffuse FDG uptake

US: Ultrasound, CT: Computed Tomography, PET: Positron Emission Tomography, FDG: fluorodeoxyglucose, C+: Contrast Enhanced.

**Table 8:** Imaging findings of ICI-related hypophysitis [70].

Imaging Modality	Findings
CT	Enlarged hypophysis
MR Necessary if suspected	Enlarged hypophysis, with convex aspect Without mass effect on the optic chiasma Thickening of the infundibulum T1w: loss of normal hyperintensity in posterior pituitary portion T1w post-contrast: homogeneous (or heterogeneous) enhancement
PET-CT (18F-FDG)	Diffuse FDG uptake

CT: Computed Tomography, PET: Positron Emission Tomography, FDG: fluorodeoxyglucose, MR: Magnetic Resonance, T1w: T1-weighted images.

**Table 9:** Differential diagnosis of hypophysitis [70].

Differential Diagnosis	Findings
Pituitary adenoma	Increased prolactin levels Asymmetric enlargement MR T1w: loss of pituitary bright spot MR T1w post-contrast: heterogeneous contrast enhancement
Pituitary metastases (melanoma, breast, and lung cancer)	Frequently involves the posterior lobe of the hypophysis Often determines diabetes insipidus (rare in cases of ctla-4 induced hypophysitis)
Lymphocytic hypophysitis	Involves young women during pregnancy or post-partum period Headache, visual impairment ACTH deficiency

T1w: T1-weighted images.

**Table 10:** Imaging findings of ICI-related endocrine pancreas injury [50].

Imaging Modality	Findings
CT MRI	Diffuse or focal autoimmune pancreatitis Enlarged “sausage-like” pancreatic enlargement, loss of pancreatic lobulation T1w post-contrast: diffuse or focal decreased pancreatic enhancement DWI: increased signal
PET-CT (18F-FDG)	Diffuse uptake

CT: Computed Tomography, MR: Magnetic Resonance, T1w: T1-weighted images, DWI: Diffusion Weighted Images, PET: Positron Emission Tomography, FDG: fluorodeoxyglucose.

**Table 11:** Imaging findings of ICI-related renal injury [80].

Imaging Modality	Findings
CT	Diffuse or focal reduction of parenchymal enhancement Cortical swelling Increase in renal size Pelvic thickening
PET-CT (18F-FDG)	Diffuse uptake

CT: Computed Tomography, PET: Positron Emission Tomography, FDG: fluorodeoxyglucose.

**Table 12:** Imaging findings of ICI-related myocarditis [26].

Imaging Modality	Findings
Echocardiography	Global left ventricular systolic dysfunction Diastolic dysfunction Regional wall motion abnormalities
Cardiac MR	Regional or global wall motion abnormalities are common but non-specific Mean value of extracellular volume fraction (ECV) higher than normal values T2w: myocardial hyperintensity compatible with edema T1w post-contrast: early enhancement due to inflammation and increased blood supply Pericardial effusion “Lake Louise criteria”
18F-FDG PET-CT	Increased myocardial FDG uptake

MR: Magnetic Resonance, T2w: T2-weighted images, T1w: T1-weighted images, PET: Positron Emission Tomography, FDG: fluorodeoxyglucose.
